# Bazhu Decoction, a Traditional Chinese Medical Formula, Ameliorates Cognitive Deficits in the 5xFAD Mouse Model of Alzheimer’s Disease

**DOI:** 10.3389/fphar.2019.01391

**Published:** 2019-11-27

**Authors:** Axiang Peng, Yuehong Gao, Xiaomei Zhuang, Yaoqi Lin, Wencan He, Yannan Wang, Wenfan Chen, Tingting Chen, Xiaoqing Huang, Renzhi Yang, Yuanpeng Huang, Shengyan Xi, Xian Zhang

**Affiliations:** ^1^Department of Traditional Chinese Medicine, Zhongshan Hospital, Xiamen University, Xiamen, China; ^2^Fujian Provincial Key Laboratory of Neurodegenerative Disease and Aging Research, Institute of Neuroscience, School of Medicine, Xiamen University, Xiamen, China; ^3^Department of Traditional Chinese Medicine, School of Medicine, Xiamen University, Xiamen, China

**Keywords:** Bazhu decoction, Alzheimer’s disease (AD), 5xFAD mice, β-amyloid (Aβ), traditional Chinese medicine (TCM)

## Abstract

Alzheimer’s disease (AD) is the most common neurodegenerative disorder associated with aging. There are currently no effective treatments for AD. Bazhu decoction (BZD), a traditional Chinese medicine (TCM) formula, has been employed clinically to alleviate AD. However, the underlying molecular mechanisms are still unclear. Here we found that middle- and high-doses of BZD ameliorated the behavioral aspects of 5xFAD transgenic mice in elevated plus maze, Y maze and Morris water maze tests. Moreover, BZD reduced the protein levels of BACE1 and PS1, resulting in a reduction of Aβ plaques. We also identified a beneficial effect of BZD on oxidative stress by attenuating MDA levels and SOD activity in the brains of 5xFAD mice. Together, these results indicate that BZD produces a dose-dependent positive effect on 5xFAD transgenic mouse model by decreasing APP processing and Aβ plaques, and by ameliorating oxidative damage. BZD may play a protective role in the cognitive and anxiety impairments and may be a complementary therapeutic option for AD.

## Introduction

Alzheimer’s disease (AD), the most common cause of dementia in the elderly, is an age-related neurodegenerative disease characterized by progressive loss of memory, cognitive dysfunction, and other executive deficits (Mattson, 2004; Stokin et al., 2005). According to the World Alzheimer Report 2018 (Patterson, 2018), 50 million people are currently affected by AD, and this number is expected to increase to more than 152 million by 2050. AD, therefore places a huge economic burden on society and on many families.

Pathologically, the classic hallmarks of AD include the accumulation of extracellular senile plaques constituted of aggregated β-amyloid (Aβ), intracellular neurofibrillary tangles (NFTs), and synapse loss (Alzheimer et al., 1995). Since Aβ is one of the primary components of amyloid plaques in the brains of patients with AD, hypotheses have been proposed regarding the key contribution of Aβ in the pathological alterations and cognitive impairment in AD (Hardy and Allsop, 1991; Hardy and Higgins, 1992). Multiple lines of evidence show that Aβ accumulation leads to neurotoxicity and memory deterioration. Aβ is generated from the proteolytic processing of amyloid precursor protein (APP) by β-secretase (BACE1) and γ-secretase (Checler, 1995; Zhang et al., 2014). Presenilin-1 (PS1) functions as the catalytic subunit of γ-secretase (Zhang et al., 2014).

Previous studies have suggested that Aβ plaques can induce activation of glial cells including astrocytes and microglia, leading to inflammation and oxidative stress (Lu et al., 2009; Li et al., 2011; Zhou et al., 2014). This may hinder neurotransmitter release, induce neuronal apoptosis and/or neuronal degeneration, and eventually contribute to cognitive impairment in AD. Malondialdehyde (MDA), a metabolite of lipid peroxidation, reflects the levels of free radicals and cellular damage (higher lipid peroxidation correlates to higher levels of MDA) (Giera et al., 2012). Superoxide dismutase (SOD) is a critical antioxidant, which can effectively eliminate the superoxide anion, protecting against neuronal toxicity (Batinic-Haberle et al., 2014).

Despite its high impact, the drugs (including donepezil HCl, rivastigmine, galanthamine, and memantine) commonly prescribed to treat AD patients merely relieve symptoms temporarily instead of decelerating the progression of disease (Wang et al., 2014; Cao et al., 2018). Preliminary clinical and preclinical studies have suggested that some traditional Chinese medicines (TCM) are beneficial to the prevention and control of AD. For instance, Fumanjian, a classic Chinese herbal formula, could ameliorate the impairment of spatial learning and memory by modulating the apoptotic signaling pathway in the hippocampus of rats with Aβ_1–40_-induced AD (Hu et al., 2014). TCM Shen-Zhi-Ling oral liquid not only improved behavioral and neuropsychological symptoms of dementia in AD patients, but also ameliorated memory impairment in AD mice by modulating the HO-1/BVR system (Xing et al., 2017). In addition, components of certain herbal formulations may constitute an alternative complementary approach to alleviate symptoms and delay the progression of AD (Jesky and Hailong, 2011; May et al., 2016). Oral administration of alpha-asarone, an active substance of Rhizoma Acori Tatarinowii (Shichangpu), improved working spatial memory in AD-like preclinical models (Limon et al., 2009). In the same study, the authors also suggested that this effect may be attributed to the protection of neuronal cells from oxidative stress, owing to decreases in NO production and Aβ-neurotoxicity. Therefore, TCM may represent an effective therapeutic option for AD, and warrants further investigation.

Bazhu decoction (BZD) is a formula of TCM that includes Radix Morindae Officinalis (Bajitian), Fructus Corni (Shanzhuyu), Pheretima (Dilong), Rhizoma Acori Tatarinowii (Shichangpu) and Arisaema cum Bile (Dannanxing). These compounds may enhance memory and ameliorate cognitive dysfunction in patients with AD (Li et al., 2016; Peng and Huang, 2017). Moreover, previous studies have suggested that water extracts from the respective herbs contained in BZD significantly improve learning and memory in AD-like animals *via* antioxidation, scavenging free radicals, neuroprotection, immunity enhancement, and by reducing the deposition of senile plaques (Jin et al., 2009; Wen and Chen, 2009; Li et al., 2013; Wang et al., 2013). Nevertheless, the mechanisms by which BZD may ameliorate AD remain elusive. In this study, we sought to explore the effect of BZD in 5xFAD mice (Oakley et al., 2006), a recognized transgenic mouse model of AD, by investigating the biological mechanisms underlying its potential therapeutic effect.

## Materials and Methods

### Experimental Animals

5xFAD transgenic mice and wild-type littermates (50% females; weight: 25 ± 3 g; age: 3 months) were from the Jackson Laboratory. 5xFAD (APP and PS1 double-transgenic) mice co-express five familial AD mutations, namely, APP K670N/M671L (Swedish), I716V (Florida), V717I (London), PS1 M146L, and L286V, and have been shown to develop major pathological features of AD more rapidly than other transgenic AD-like animal models (Oakley et al., 2006). These animals present with increased amyloid plaque deposits and memory impairments in the Y and Morris water mazes at the ages of 2 and 4 months, respectively (Oakley et al., 2006; Wang et al., 2014). All animals were fed, cared for, and handled in accordance with the Guide for the Care and Use of Laboratory Animals of the Xiamen University, and the Animal Ethics Committee Guidelines of the Animal Facility of the Zhongshan Hospital Xiamen University. The animals were acclimatized to the facilities for one week before the beginning of the treatment.

### Drug Preparation and Reagents

Chinese medicines used for the concoction of BZD were supplied by the Zhongshan Hospital Xiamen University (Xiamen, China). Each herb was identified by the experts in the School of Pharmaceutical Sciences of Xiamen University. All voucher specimens were deposited at the Xiamen Botanical Garden (http://sweetgum.nybg.org/science/ih/herbarium-details/?irn=249232) (Herbarium Code: XMBG) for future reference. BZD containing Radix Morindae Officinalis, Fructus Corni, Pheretima, Rhizoma Acori Tatarinowii, and Arisaema cum Bile (3:2:3:3:2) (see **Table 1**) was strictly decocted in accordance with the standards of Chinese medicine, and 3 concentrations (0.211 g/ml, 0.423 g/ml, and 0.845 g/ml) were prepared using a heat cycle oven. Additionally, donepezil HCl (Aricept) was purchased from Eisai pharmaceutical Co., Ltd. Donepezil HCl was dissolved in a 1% solution of sodium carboxymethyl cellulose to obtain a concentration of 0.0325 mg/ml.

**Table 1:** Information of components in BZD.

Chinese name	Botanical name	Common name	Weight (g)	Voucher numbers	Part used
Ba Ji Tian	*Morinda officinalis* How	Radix Morindae Officinalis	15	180901	Root
Shan Zhu Yu	*Cornus officinalis* Sieb. et Zucc.	Asiatic Cornelian Cherry Fruit	10	180221	Matured sarcocarp
Shi Chang Pu	*Acorus tatarinowii* Schott	Grassleaf Sweetflag Rhizome	15	180901	Rhizome
Di Long	*Pheretima aspergillum* (E. Perrier), *Pheretima vulgaris* Chen, *Pheretima guillelmi* (Michaelsen) or *Pheretima Pectinifera* Michaelsen	Earth Worm	15	181121	Dried body
Dan Nan Xing	*Arisaema erubescens* (Wall.) Schott, *Arisaema heterophyllum* B1. or *Arisaema amurense* Maxim	Arisaema Cum Bile	10	171203	Powder

Antibodies against APP (369) and PS1-NTF (Ab14) were generated in-house (Thinakaran et al., 1996; Xu et al., 1997). Anti-BACE1 (3D5) antibody was kindly provided by Professor Robert Vassar (Northwestern University). Anti-β-actin antibody was purchased from ZEN bioscience, and anti-β-amyloid antibody was obtained from Abcam. Horseradish peroxidase labeled secondary goat anti-rat IgG antibody and goat anti-rabbit IgG antibody were purchased from Pierce, and polyvinylidene difluoride (PVDF) membranes were purchased from Millipore. The protein loading marker was purchased from Fermentas, and the protease inhibitors were purchased from Roche. X-ray blue films were purchased from Kodak; BSA and TEMED were purchased from Sigma.

### UHPLC-MS

The major chemical constituents of BZD were profiled by ultra-high performance liquid chromatography (UHPLC) coupled with a high resolution electrospray ionization mass (HR-ESI-MS) detector. 10 mg lyophilized powder was dissolved in 1 mL of ultrapure water through ultrasonic method. The solution was filtered with 0.22 µm nylon filter membrane before injection into the UHPLC. The UHPLC separation was performed over a C18Kinetex column (100 × 2.1 mm i.d., 2.6 µm, Phenomenex Inc., Torrance, USA) at 35 °C on the Thermo UltiMate 3000 LC system (Thermo Fisher Scientific, Bremen, Germany). The mobile phases were acetonitrile (A) and 0.1% formic acid with water (*v/v*) (B). Samples were eluted by gradients according to the elution program as follows: A from 5% to 35% and B from 95% to 65% during 0–30 min, A from 35 to 100% and B from 65 to 0% during 30–35 min, A and B were kept at 100 and 0% respectively during 35–45 min. The column was maintained at 35 °C and eluted at a flow rate of 0.3 mL/min. The injected volume was 5 µL. A diode array detector with detection wavelength of 254 nm, and a high resolution ESI-MS detector were used to record the HPLC chromatograms. After UHPLC, samples were analyzed by MS spectra on a Thermo Q-Exactive system. The mass spectrometer with positive and negative ionizations was calibrated across *m/z* 100–1,500 using the manufacturer’s calibration standards mixture (caffeine, MRFA and Ultramark 1621 in anacetonitrile–methanol–water solution containing 1% acetic acid) allowing mass fluctuation of no more than 5 ppm in the external calibration mode. The ionization voltage was 3.5 kV, and the capillary temperature was set at 300 °C.

### Grouping and Treatment

The 5xFAD mice were randomly allocated to 5 groups, namely, control (5xFAD-Control), low-dose BZD (5xFAD-BZD-L), medium-dose BZD (5xFAD-BZD-M), high-dose BZD (5xFAD-BZD-H), and donepezil HCl (5xFAD-Donep) (n = 8/group). Wild-type littermates, used as control mice, were randomly allocated to control (WT-Control), BZD (WT-BZD), and donepezil HCl (WT-Donep) groups (n = 8/group).

The clinical dosing of BZD and Donepezil HCl was determined in accordance with the Pharmacopoeia of the People’s Republic of China (2015). Mice in the control groups were administered 20 mL/(kg·d) double distilled water by gavage. The 5xFAD-Donep and WT-Donep mice were administered 20 mL/(kg·d) donepezil HCl at a dose of 0.65 mg/(kg·d) and concentration of 0.0325 mg/mL. In addition, mice in the 5xFAD-BZD-L, 5xFAD-BZD-M, 5xFAD-BZD-M, and WT-BZD groups were orally administered 20 mL/(kg·d) BZD at doses of 4,225 g/(kg·d), 8,450 g/(kg·d), 16,900 g/(kg·d), and 16,900 g/(kg·d), respectively. All mice received treatment once daily by gavage for 12 weeks.

### Behavioral Tests

The behavioral tests were initiated 10 days before the end of treatment (n = 8/group). On a normal test day, mice were allowed to adapt to the experimental environment for 30 min. Only one behavioral test was performed on each day. The order of the tests was organized so that easy to difficult tests could be performed in standard room-lighting conditions. Mazes were cleaned with 75% alcohol immediately after the end of the test to minimize scent trails. Smart 3.0 software, a data collection system, and an overhead camera were used to monitor the activity of the mice.

The methods for the open field test and elevated plus maze used in our study have been described in detail elsewhere (Liu et al., 2014). For the open field test, mice were placed in an arena measuring 40 cm × 40 cm × 40 cm divided into 16 squares, for 10 min. The total distance moved in the arena and the time spent in the central arena (central 4 squares) were analyzed for each mouse. For the elevated plus maze, the apparatus, which was elevated 40 cm above the ground, consisted of two opposing open arms (30 cm × 6 cm) and two opposing closed arms (30 cm 6 cm × 15 cm) with an open roof. Each mouse was placed at the center of the maze facing an open arm. Then the experimenter exited the room, allowing the mouse to freely explore for 5 min. The frequencies of entries to the open arms, and the time spent in the open arms were recorded as an index of anxiety (higher index, lower anxiety).

Study has shown that (Ohno et al., 2004), the procedure for the Y Maze did not involve any training, reward, or punishment and allowed researchers to evaluate the rodents’ spontaneous spatial working memory. This test mostly assesses the function of the hippocampus. The Y maze comprised 3 symmetrical arms (30 cm × 6 cm × 15 cm) separated by 120 degrees. Every mouse was placed in the center of Y maze, with the head in the same direction, and was allowed to explore the arms freely for 5 min. The sequence and total number of arm entries were recorded. Data were analyzed using the percentage of alternation triplet, which was the number of triads containing entries into all 3 arms divided by the maximum possible number of alternations (the total number of arms entered - 2) × 100.

For the Morris water maze, the task was carried out in strict accordance with the protocol described by Geda (Vorhees and Williams, 2006). The maze consisted of a 1.2 m diameter, light blue, plastic, circular pool filled with water to a depth of 31 cm with a temperature of 22 ± 1°C. The maze was divided into four quadrants (SW, SE, NE, and WN). A transparent platform was placed at the SW quadrant and was submerged ∼1 cm beneath the surface of the water. The test included two phases: spatial navigation training and probe experiments. During spatial navigation training, mice were first placed in one of the four starting locations facing the pool wall, and were allowed to swim until finding the platform in 60 s. The time that each mouse took to find the hidden platform was recorded. If the mice did not find the platform within 60 s, they were guided to the platform by the experimenter and were allowed to remain at the platform for 10 s. For the probe experiments, each trial was conducted when the animal’s latency to reach the platform tended to stabilize. The hidden platform was removed, and mice were placed in the pool at the opposite quadrant of the platform, and were allowed to swim freely for 60 s. Time spent in each quadrant, frequencies of crossings to the previous location of the target platform, and motion tracings were obtained using an automated video tracking software from Smart 3.0.

### Collection of Brain Tissues

Experimental mice were sacrificed after completing the Morris water maze test. Briefly, the animals were anesthetized with 1.5% pentobarbital sodium, perfused with phosphate buffered saline (PBS), and the brains were rapidly harvested on ice. Six mice were randomly selected from each group, and the left hemi-brains were fixed in fresh 4% paraformaldehyde and were used for immunohistochemistry analysis. The right hemi-brains were immediately frozen in liquid nitrogen for storage until homogenization for other biochemical assays.

### Immunohistochemistry

Immunohistochemistry was performed as previously described (Hu et al., 2015) (n = 6/group). In summary, the brains were fixed for 48 h at 4°C in 4% paraformaldehyde and were then transferred to 30% sucrose for 2–3 days. O.C.T., a tissue freezing medium, was employed to freeze the brains and the tissue was then sectioned into 10-µm slices using a freezing microtome. Immunohistochemistry staining was used to determine Aβ plaques in the cortex and hippocampus. Anti-β-amyloid antibody (ab126649) was employed at a concentration of 1:500. The signal was revealed using the DAB chromogenic reagent kit. Light microscopy was used to capture the images. Densitometry analysis of the images was conducted using Image Pro Plus 6.0 software.

### Oxidative Stress Assay

In summary, the hippocampal and cortical tissues were separated from the right hemi-brains (n = 6/group), homogenized in phosphate buffered saline (PBS) (mass ratio of 1:1) and were then divided into two parts, one for detecting SOD/MDA, and the other for detecting protein by western blot analysis. PBS (mass ratio of 1:10) was added to the homogenates and homogenization was repeated using a Polytron homogenizer. The homogenates were centrifuged at 3,000 r/min for 10 min at 4°C and the supernatants were collected. The SOD activity and MDA levels were determined according to the manufacturers’ instructions for Total Superoxide Dismutase (T-SOD) assay kit (Hydroxylamine method) or Malondialdehyde (MDA) assay kit (TBA method) from Nanjing Jiancheng Bioengineering Institute.

### Western Blot

The other part of homogenates from hippocampal and cortical tissues (n = 6/group) was homogenized again in a buffer solution containing a protease inhibitor mix (Roche). Samples were centrifuged at 12,000g for 20 min at 4°C. The supernatant was collected and the proteins in the final elute were quantified using a BCA protein assay kit according to the manufacturer’s protocol. Equivalent amounts of protein were separated in 10% sodium dodecyl sulphate polyacrylamide gel (SDS-PAGE) and were then electrophoretically transferred to a PVDF membrane. The membranes were blocked with Tris-buffered saline containing 0.05% Tween-20 (TBST) and 5% skimmed milk for 2 h. The membranes were then probed with primary antibodies (anti-APP, anti-PS1-NTF and anti-BACE1) overnight at 4 °C, followed by incubation with horseradish peroxidase-linked secondary antibodies at room temperature (23 ± 2 °C) for 1 h. Signals were revealed using enhanced chemiluminescence western blotting detection reagents. Relative protein levels were normalized to β-actin. The intensity of the immunoreactive bands was quantified using Image J software.

### Statistical Analysis

Data have been expressed as mean ± standard deviation (SD). Statistical analyses were performed using GraphPad Prism 7.0 (GraphPad Software Inc., La Jolla, United States). One-way analyses of variance (One-Way ANOVA) was used to compare the means of multiple groups. The least significant difference (LSD) method was selected for *post hoc* analysis. *P* < 0.05 was considered statistically significant.

## Results

### Analysis of BZD

**Figure 1:**
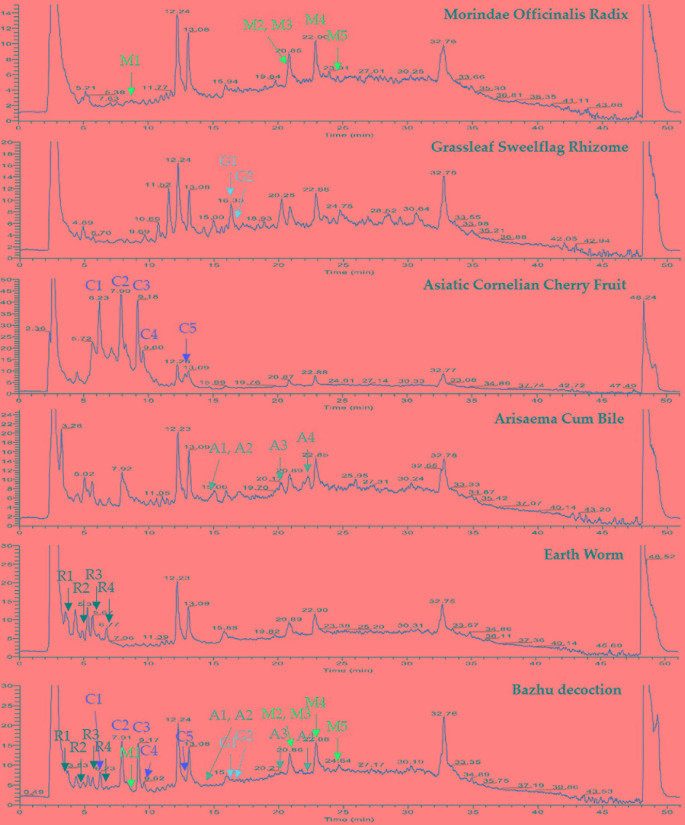
UHPLC-MS chemical fingerprint of BZD. Asperuloside (M1), rubiadin (M2), physcion (M3), rubiadin-1-methyl ether (M4), 1,6-dihydroxy-2-methoxyanthraquinone (M5), 2,4,5-trimethoxybenzaldehyde (G1), β-asarone (G2), 5-hydroxymethyl-2-furaldehyde (C1), morroniside (C2), loganin (C3), sweroside (C4), cornuside (C5), taurochenodeoxycholic acid (A1), glycohyodeoxycholic acid (A2), cholic acid (A3), hyodeoxycholic acid (A4), leucine (R1), adenine (R2), phenylalanine (R3), and tryptophan (R4).

**Figure 2:**
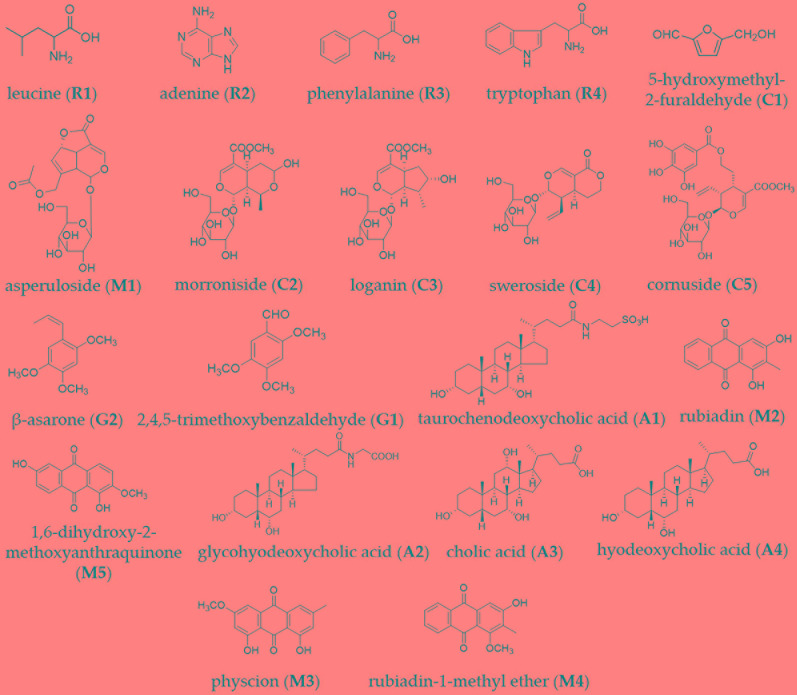
Chemical structure of the major identified components in BZD by UHPLC-MS: leucine, adenine, phenylalanine, tryptophan, 5-hydroxymethyl-2-furaldehyde, asperuloside, morroniside, loganin, sweroside, cornuside, β-asarone, 2,4,5-trimethoxybenzaldehyde, taurochenodeoxycholic acid, rubiadin, 1,6-dihydroxy-2-methoxyanthraquinone, glycohyodeoxycholic acid, cholic acid, hyodeoxycholic acid, physcion and rubiadin-1-methyl ether.

BZD was separated using the UHPLC-MS system and its chromatographic fingerprinting established. Comparing the retention time, UV and MS spectra with reference samples, the following twenty major components were identified: leucine (peak R1, Rt = 3.76 min), adenine (peak R2, Rt = 4.92 min), phenylalanine (peak R3, Rt = 5.27 min), 5-hydroxymethyl-2-furaldehyde (peak C1, Rt = 6.23 min), tryptophan (peak R4, Rt = 6.76 min), morroniside (peak C2, Rt = 7.96 min), asperuloside (peak M1, Rt = 8.82 min), loganin (peak C3, Rt = 9.41 min), sweroside (peak C4, Rt = 9.60 min), cornuside (peak C5, Rt = 12.81 min), taurochenodeoxycholic acid (peak A1, Rt = 14.43 min), glycohyodeoxycholic acid (peak A2, Rt = 14.85 min), 2,4,5-trimethoxybenzaldehyde (peak G1, Rt = 16.31 min), β-asarone (peak G2, Rt = 16.99 min), cholic acid (peak A3, Rt = 20.21 min), rubiadin (peak M2, Rt = 20.86 min), physcion (peak M3, Rt = 21.31 min), hyodeoxycholic acid (peak A4, Rt = 22.28 min), rubiadin-1-methyl ether (peak M4, Rt = 22.94 min), and 1,6-dihydroxy-2-methoxyanthraquinone (peak M5, Rt = 24.21 min) as shown in **Figures 1** and **2**. Detailed information of BZD’s components identified by UHPLC is provided in the **Supplementary Table**.

### BZD Improves Learning and Memory Abilities and Alleviated Anxiety-Related Behaviors in 5xFAD Mice

**Figure 3:**
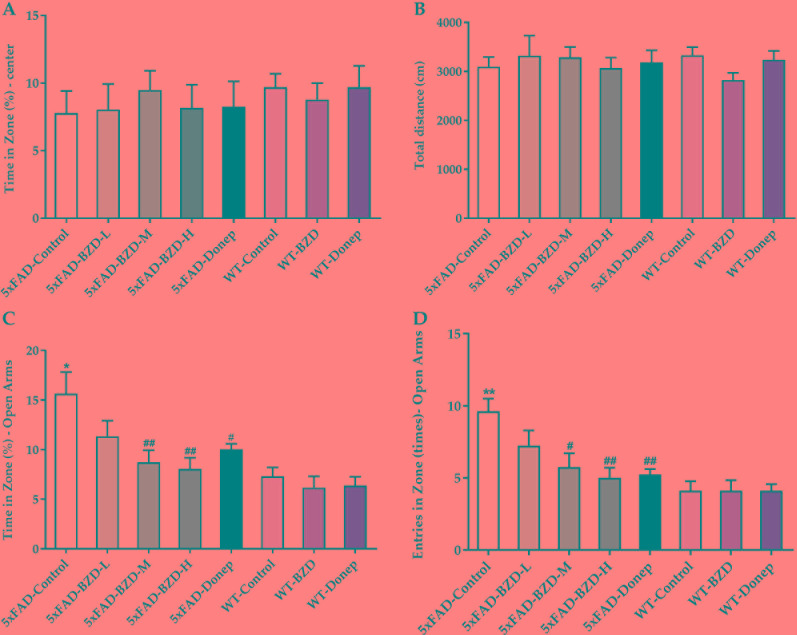
BZD alleviates anxiety-related behaviors in 5xFAD mice. **(A**, **B)** Mice (5xFAD-Control, 5xFAD-BZD-L, 5xFAD-BZD-M, 5xFAD-BZD-H, 5xFAD-Donep, WT-Control, WT-BZD and WT-Donep) were analyzed for duration in the center **(A)** and total travel distance **(B)** in the open field test. Data are expressed as the mean ± SD from 8 mice per group, not significant, One-Way ANOVA. **(C**, **D)** Mice (5xFAD-Control, 5xFAD-BZD-L, 5xFAD-BZD-M, 5xFAD-BZD-H, 5xFAD-Donep, WT-Control, WT-BZD and WT-Donep) were analyzed for time in zone – open arms **(C)** and entries in zone – open arms **(D)** in elevated plus maze. Data are expressed as the mean ± SD from 8 mice per group. *P < 0.05, ** P < 0.01 compared with WT-Control group; ^#^*P* < 0.05, ^##^*P* < 0.01 compared with 5xFAD-Control group, One-Way ANOVA.

To evaluate the impact of BZD treatment on locomotor activity and anxiety-related behaviors, we performed open field and elevated plus maze tests. In the open field test, there was no significant difference in the percentage of time spent in the center of the arena, or in the total distance traveled among groups (*P* > 0.05) (**Figures 3A, B**), indicating that locomotor activity of 6-month-old 5xFAD mice is not altered. In the elevated plus maze, the 5xFAD-Control mice spent greater time in the open arms and had a larger number of entries in the open arms (*P* < 0.01) (**Figures 3C, D**) than the mice in the WT-Control group, suggesting that at 6-months, 5xFAD mice showed attenuated anxiety in dangerous environments, with enhanced behavioral disinhibition (Olausson et al., 1999). BZD or donepezil HCl had no effect on anxiety in wild-type mice. Interestingly, we observed that the 5xFAD-BZD-M, 5xFAD-BZD-H, and 5xFAD-Donep groups exhibited a marked decrease in the percentage of time spent in the open arms, and in the frequency of entries into the open arms when compared to the 5xFAD-control group (*P* < 0.05 or *P* < 0.01) (**Figures 3C, D**), confirming that medium and high doses of BZD attenuated the behavioral disinhibition of 5xFAD mice.

**Figure 4:**
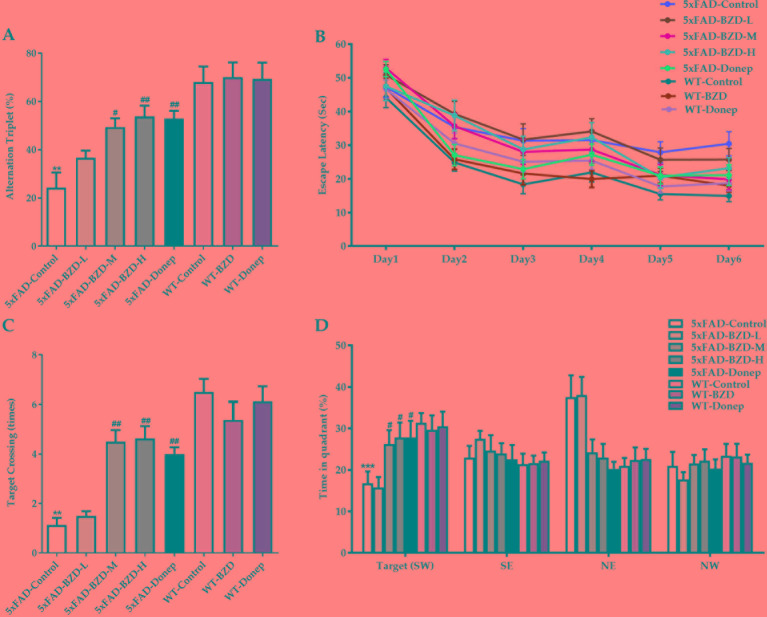
BZD improves learning and memory abilities in 5xFAD mice. **(A)** Mice (5xFAD-Control, 5xFAD-BZD-L, 5xFAD-BZD-M, 5xFAD-BZD-H, 5xFAD-Donep, WT-Control, WT-BZD and WT-Donep) were analyzed for spontaneous alternation behavior by Y-maze tests. Data are expressed as the mean ± SD from 8 mice per group. **P < 0.01 compared with WT-Control group; ^#^*P* < 0.05, ^##^*P* < 0.01 compared with 5xFAD-Control group, One-Way ANOVA. **(B**–**D)** Mice (5xFAD-Control, 5xFAD-BZD-L, 5xFAD-BZD-M, 5xFAD-BZD-H, 5xFAD-Donep, WT-Control, WT-BZD and WT-Donep) were analyzed for spatial learning and memory in Morris water maze tests. Mice were analyzed for escape latency within a 6-day training period **(B)**. On the 7th day, mice were assayed for the number of crossings over the target platform region **(C)** and time spent in target quadrant **(D)**. Data are expressed as the mean ± SD from 8 mice per group. **P < 0.01, ***P < 0.001 compared with WT-Control group; ^#^*P* < 0.05, ^##^*P* < 0.01 compared with 5xFAD-Control group, One-Way ANOVA.

To investigate the effects of BZD on the pathological features related to learning and memory, we also tested the animals in the Y and Morris water mazes. In the Y maze, the 5xFAD-Control group showed a pronounced decrease in the percentage of triple alternations compared to the WT-Control group (*P* < 0.01) (**Figure 4A**), suggesting that spontaneous spatial working memory is impaired in 5xFAD mice. BZD and Donepezil increased the percentage of triple alternations in 5xFAD mice (*P* < 0.05) (**Figure 4A**), suggesting that medium and high doses of BZD improved spontaneous spatial working memory in 5xFAD mice. In the Morris water maze, as anticipated, we found that training shortened the escape latency time (ELT) of mice across groups (**Figure 4B**). On the sixth day of training, the mean ELT of the wild-type mice was under 20 s, while the 5xFAD mice presented with values of 20 s or higher. Nevertheless, both wild-type and 5xFAD mice presented with stable values of ELT in the last two training days, suggesting that the probe experiment could be conducted on the seventh day. During the probe trials, the 5xFAD-Control mice showed a dramatic decrease in spatial learning and memory compared to wild-type mice (*P* < 0.01 or *P* < 0.001) (**Figures 4C, D**). Medium- and high-dose BZD and donepezil reduced this deficit, as demonstrated by the increased number of target (platform) crossing events and the percentage of time spent in the target quadrant, compared to the 5xFAD-control group (*P* < 0.05 or *P* < 0.01) (**Figures 4C, D**). Thus, Medium and high doses of BZD improved spatial learning and memory in 5xFAD mice.

### BZD Reduces Aβ Deposition

**Figure 5:**
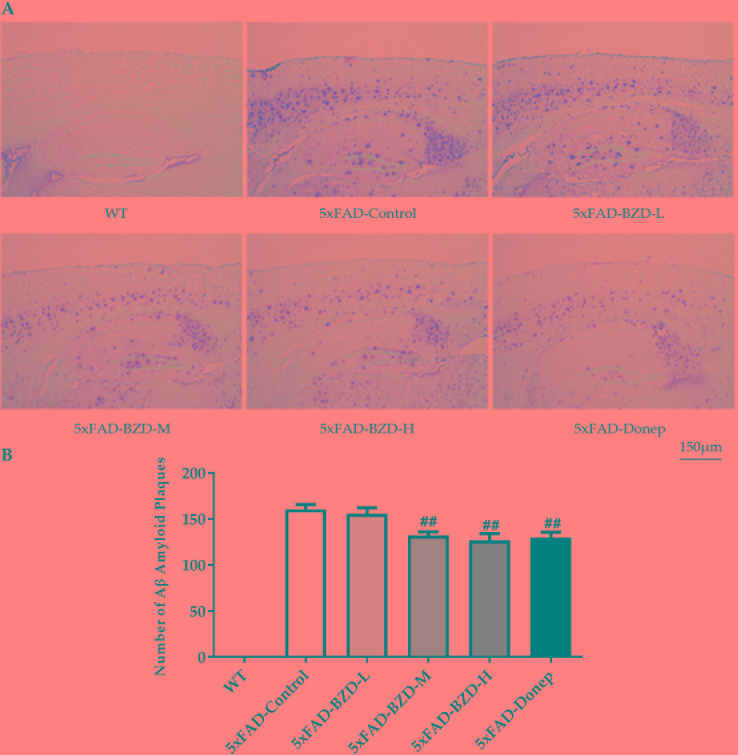
BZD Reduces Aβ Deposition. **(A)** Immunohistochemistry analysis of Aβ plaques in the cortex and hippocampus of 5xFAD Mice (5xFAD-Control, 5xFAD-BZD-L, 5xFAD-BZD-M, 5xFAD-BZD-H and 5xFAD-Donep) and wild type (WT) littermates. Representative images of Aβ plaques; Scale bar, 150 µm. **(B)** Quantification of Aβ plaques in A. Data are expressed as the mean ± SD from 6 mice per group. ^##^*P* < 0.01 compared with 5xFAD-Control group, One-Way ANOVA.

Cumulative evidence suggests that aggregation of Aβ in the brain is a causative factor for AD pathogenesis. Aβ plaques were determined in the cortex and hippocampus of 5xFAD Mice and wild type littermates by immunostaining. The results showed that no positive staining was observed in the cortex or hippocampus of wild-type mice, whereas the 5xFAD mice presented with a high number of Aβ plaques (**Figure 5A**). Low-dose BZD did not affect the quantity of Aβ plaques (*P* > 0.05) (**Figure 5B**). However, donepezil HCl and medium- and high-dose BZD significantly reduced the burden of Aβ plaques in 5xFAD mice (*P* < 0.01) (**Figure 5B**).

### BZD Modulates APP Processing

**Figure 6:**
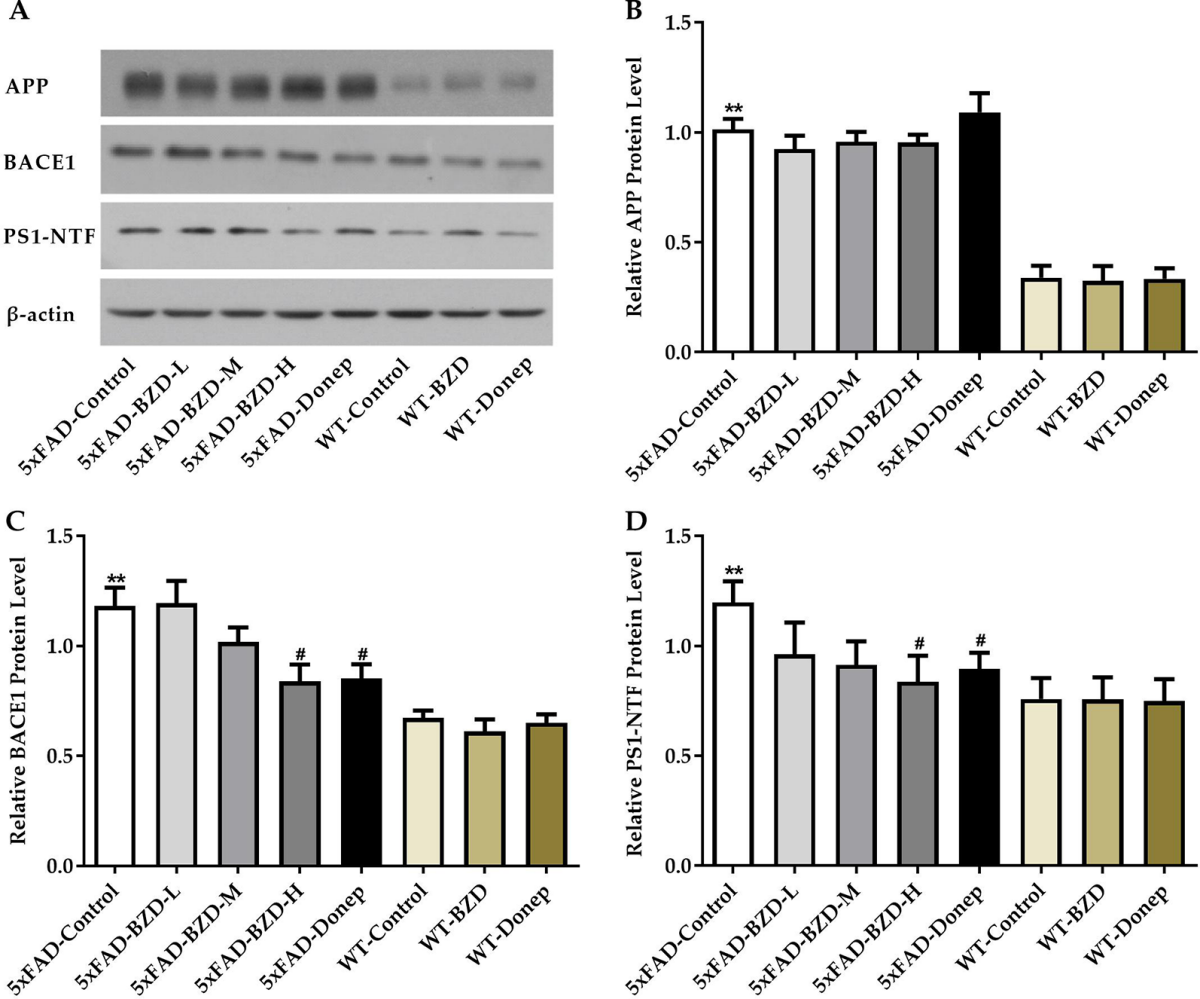
BZD Modulates APP Processing. **(A)** Equal protein quantities of lysates from hippocampal and cortical tissues of mice (5xFAD-Control, 5xFAD-BZD-L, 5xFAD-BZD-M, 5xFAD-BZD-H, 5xFAD-Donep, WT-Control, WT-BZD and WT-Donep) were subjected to western blot with antibodies against APP, BACE1, PS1-NTF, and β-actin. APP **(B)**, BACE1 **(C)** and PS1-NTF **(D)** protein intensities were quantified by densitometry, and normalized to respective β-actin levels. Data are expressed as the mean ± SD from 6 mice per group. **P < 0.01 compared with WT-Control group; ^#^*P* < 0.05 compared with 5xFAD-Control group, One-Way ANOVA.

β-secretase and γ-secretase are two key elements of the amyloidogenic pathway of APP processing associated with the production of Aβ. To investigate whether BZD could affect APP processing, we checked the protein levels of APP, BACE1 and PS1-NTF in the lysates from hippocampal and cortical tissues of the experimental mice (5xFAD-Control, 5xFAD-BZD-L, 5xFAD-BZD-M, 5xFAD-BZD-H, 5xFAD-Donep, WT-Control, WT-BZD and WT-Donep) (**Figure 6A**). As expected, 5xFAD mice presented with significantly higher proteins levels of APP, BACE1 and PS1-NTF compared to the wild-type mice (**Figures 6B–D**). BZD and donepezil had no effect on APP protein levels (*P* > 0.05) (**Figure 6B**). High-dose BZD and donepezil decreased BACE1 protein levels in 5xFAD mice (*P* < 0.05); and medium and low-dose BZD had no effect (**Figure 6C**). PS1-NTF is the catalytic subunit of γ-secretase, an enzyme that plays an important role during APP processing. We found that Donepezil HCl and high but not medium or low doses of BZD, reduced the levels of PS1-NTF (*P* < 0.05) (**Figure 6D**). These results indicate that BZD may reduce Aβ level by modulating APP processing.

### BZD Decreases Oxidative Damage in the Cortex and Hippocampus of 5xFAD Mice

**Figure 7:**
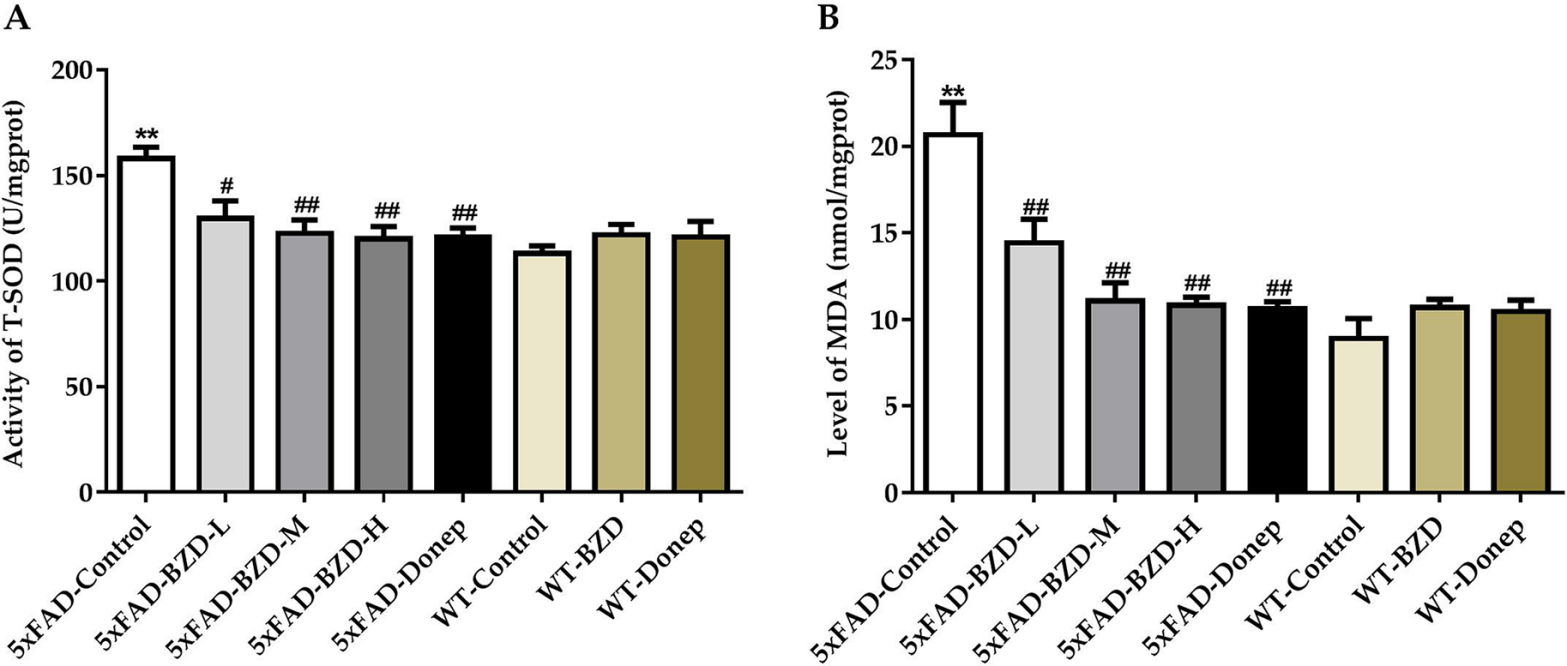
BZD Decreases MDA Levels and SOD Activity in the Cortex and Hippocampus of 5xFAD Mice. **(A)** Activities of SOD were measured in the cortical and hippocampal tissue samples of mice (5xFAD-Control, 5xFAD-BZD-L, 5xFAD-BZD-M, 5xFAD-BZD-H, 5xFAD-Donep, WT-Control, WT-BZD and WT-Donep). Data are expressed as the mean ± SD from 6 mice per group. **P < 0.01 compared with WT-Control group; ^##^*P* < 0.05, ^##^*P* < 0.01 compared with 5xFAD-Control group, One-Way ANOVA. **(B)** MDA levels were measured in the cortical and hippocampal tissue samples of mice (5xFAD-Control, 5xFAD-BZD-L, 5xFAD-BZD-M, 5xFAD-BZD-H, 5xFAD-Donep, WT-Control, WT-BZD and WT-Donep). Data are expressed as the mean ± SD from 6 mice per group. **P < 0.01 compared with WT-Control group; ^##^*P* < 0.01 compared with 5xFAD-Control group, One-Way ANOVA.

Oxidative stress is implicated in the pathogenesis of AD. Recent studies have shown that increases in MDA content and the fluctuant activity of SOD are associated with the aggregation of Aβ plaques and AD (Zhou et al., 2014). To explore whether BZD could have an impact on MDA/SOD, we quantified MDA levels and SOD activity in homogenates of the cortical and hippocampal tissue samples. 5xFAD mice presented with higher levels of MDA and greater SOD activity than those in the WT-Control group (*P* < 0.01) (**Figures 7A, B**), suggesting that 5xFAD mice present with higher levels of free radicals and cellular damage. Interestingly, high- and medium-dose BZD and donepezil HCl reduced both MDA levels and SOD activity (*P* < 0.05) (**Figures 7A, B**). These results indicate that BZD decreases oxidative damage in the cortex and hippocampus of 5xFAD Mice.

## Discussion

In this study, we evaluated the potential impact of BZD, an alternative Chinese medicine formulation, on behavioral alterations in a transgenic mouse model of AD, including its key molecular hallmarks. We found that BZD exerted a dose-dependent beneficial effect in 5xFAD mice. Medium and high doses of BZD not only ameliorated behaviors related to the disease, but also reduced the production and aggregation of Aβ plaques, probably by reducing the levels of BACE1 and PS1. We also identified a beneficial effect on oxidative stress (SOD/MDA) in the hippocampi of 5xFAD mice. We suggest that BZD represent a therapeutic option for AD patients that is worth to be explored in randomized clinical studies.

According to the theory of TCM, AD results from insufficiency of kidney essence, intermingling of phlegm and blood, and fluid stasis (Peng and Huang, 2017). AD may, therefore, benefit from treatments invigorating the qi of the kidney, stimulating elimination of phlegm and promoting blood circulation to remove obstruction and dredge collaterals (Zhu and Hu, 2007). Based on this assumption, Morinda officinalis How. (Bajitian), Cornus officinalis Sieb. et Zucc. (Shanzhuyu), Earthworm (Dilong), Acori Tatarinowii Rhizoma (Shichangpu), and Arisaema cum Bile (Dannanxing) were selected to compound the BZD formula. Bajitian is the sovereign drug that warms and recuperates the yang of the kidney, Shanzhuyu is the minister drug, nourishing and invigorating the yin and qi of the kidney, respectively, Shichangpu and Dannanxing are assistant drugs, that increase the elimination of phlegm, and Dilong is the envoy drug, which not only promotes blood circulation and removes obstruction in the collaterals, but also assists other drugs to penetrate into the diseased areas (Li et al., 2016; Peng and Huang, 2017). Therefore, BZD is consistent with the TCM prescription theory for AD, which aims to adjust the patients’ habitus including the syndromes of intermingled deficiency and excess, heat and cold, or phlegm and blood stasis. BZD may optimize the rapid aging internal environment and mobilize immune function, thereby extending life and improving intelligence. An increasing number of studies have suggested that single components of BZD are beneficial for AD. Oligosaccharides from Radix Morindae Officinalis improve memory function of AD animal models (Yang et al., 2018). Isolated compounds from Radix Morindae Officinalis ameliorate AD (Lee et al., 2017). Other pharmacological studies have also demonstrated that extracts of Radix Morindae Officinalis have anti-Alzheimer’s, anti-fatigue, anti-aging, cardiovascular protective, anti-oxidative, immune-regulatory, and anti-inflammatory activities (Zhang et al., 2018). Studies using Fructus Corni have suggested a wide range of therapeutic properties for this compound, including neuroprotection, anti-amnesia, anti-aging, anti-inflammation, analgesia, antioxidation, anti-osteoporosis, and immunoregulation (Huang et al., 2018a). Rhizoma Acori Tatarinowii reduces amyloid plaques and tau phosphorylation, and has anti-inflammatory effects (Deng et al., 2015; Song et al., 2018). Therefore, the anti-inflammatory and immunity-enhancing effects may contribute to its therapeutic effects in AD. Arisaema cum Bile is useful for treating diseases associated with oxidative imbalance, such as AD, Parkinson’s disease, heart disease, rheumatoid arthritis and diabetes (Ahn et al., 2012). Earthworm may have immune regulatory, anti-inflammatory, anti-bacterial and anti-oxidative properties, which are closely related to AD (Huang et al., 2018b). BZD therapeutic effects are likely to benefit from the synergistic effects of these compounds when administered concomitantly. However, empirical evidence supporting this hypothesis is still scarce.

In this study, BZD was separated using the UHPLC-MS system and 20 major chemical components were identified. Taurochenodeoxycholic acid, as a signaling molecule, shows obvious anti-inflammatory and immune regulation properties. Taurochenodeoxycholic acid plays a role in the PKC/JNK signaling pathway, and may be a latent effective pharmaceutical product for apoptosis- or age-related diseases (Wang et al., 2016). Tryptophan is converted *via* the 5-hydroxyindole pathway to serotonin, a neurotransmitter in the brain, paracrine and endocrine signal in the gut, and a paracrine signal and vasoconstrictor released by platelets. It is also converted by endocrine and other cells *via* serotonin to the hormone melatonin, which may improve mood and sleep (Fernstrom, 2016). Rubiadin-1-methyl ether may be a therapeutic candidate for bone diseases characterized by enhanced bone resorption (He et al., 2018a). Asperuloside and sweroside both can exert an anti-inflammatory effect *via* suppression of the NF-κB signaling pathways in LPS-induced RAW 264.7 cells (He et al., 2018b; Wang et al., 2019). Rubiadin, a dihydroxy anthraquinone, isolated from alcoholic extract of Rubia cordifolia, possesses potent antioxidant property (Tripathi et al., 1997). Hyodeoxycholic acid protects the neurovascular unit against oxygen-glucose deprivation and reoxygenation-induced injury *in vitro* (Li et al., 2019). 2,4,5-trimethoxybenzaldehyde, a bitter principle in plants, suppresses adipogenesis through the regulation of ERK1 (Wang and Kuo, 2014). Physcion blocks cell cycle and induces apoptosis in human B cell precursor acute lymphoblastic leukemia cells by downregulating HOXA5 (Gao et al., 2017). Morroniside protects against cerebral ischemia/reperfusion injury by inhibiting neuron apoptosis and MMP2/9 expression (Zeng et al., 2018). Loganin exerts sedative and hypnotic effects *via* modulation of the serotonergic system and GABAergic neurons (Shi et al., 2019). Cornuside attenuates apoptosis and ameliorates mitochondrial energy metabolism in rat cortical neurons (Jiang et al., 2009; Jiang et al., 2014). Cholic acid can be used as a treatment for cerebrotendinous xanthomatosis in adults (Mandia et al., 2019). 5-hydroxymethyl-2-furaldehyde (5-HMF) may block scopolamine-induced learning deficit and enhance cognitive function *via* the activation of NMDA receptor signaling, including CaMKII and ERK, and would be an effective candidate against cognitive disorders, such as Alzheimer’s disease (Lee et al., 2015). Leucine-rich repeat kinase 2 is associated with activation of the paraventricular nucleus of the hypothalamus and stress-related gastrointestinal dysmotility (Maekawa et al., 2019). All of these chemical components have anti-aging, anti-inflammatory, anti-oxidative, immune-regulatory, anti-tumor, and neuroregulatory properties, among others.

AD is characterized by memory loss and cognitive impairment, and various neuropsychiatric symptoms including depression, anxiety, hallucination, irritability, indifference and disinhibition (Geda et al., 2013). Here the potential effects of BZD on locomotor activity and anxiety were assessed by the open field and elevated plus maze tests; while the effect of BZD on learning and memory was assessed using the Y and Morris water maze tests. The open field test reflects locomotor activity, spontaneous exploration, and anxiety levels of mice in a novel environment (typically, the greater is the time spent in the central arena, the less is the anxiety; greater distances traveled are associated with greater locomotor activity) (Heredia et al., 2014). In this cohort, 6-month-old 5xFAD mice and the wild-type littermates did not differ with respect to general locomotor activity. BZD had no effect on the locomotor activity of 5xFAD mice.

To further assess the effects o on anxiety levels, we used the elevated plus maze. Since mice have an innate fear of elevated open places, when allowed to freely explore the maze, they typically enter less and spend shorter time in the open arms as compared to the closed arms of the maze (Walf and Frye, 2007). Therefore, a greater number of entries and time spent in the open arms indicate low anxiety levels and behavioral disinhibition (Walf and Frye, 2007). Moreover, 6-month-old 5xFAD mice presented with lower anxiety in dangerous environments, indicating a tendency toward disinhibition. This result concurs with that of previous reports suggesting that approximately 35% of mild AD patient’s exhibit disinhibition, which manifests as impulsive behavior, talking to strangers like acquaintances, ignoring dangers, and excessive euphoria (Olausson et al., 1999; Starkstein et al., 2004). Importantly, medium and high doses of BZD attenuated this behavioral alteration by increasing vigilance and attenuating behavioral disinhibition in the 5xFAD mice.

The Y maze is typically used to assess spontaneous spatial working memory in rodents (Ohno et al., 2004) by analyzing the percentage of alternation triplet (the higher the percentage of alternation triplets, the better the spontaneous spatial working memory). In our study, 5xFAD mice exhibited impaired spontaneous spatial working memory. We found that medium and high doses of BZD improved memory impairments in 5xFAD mice, as shown by an increase in the percentage of alternation triplets. The Morris water maze is one of the classic behavioral tests for spatial learning and memory capacity in rodents (Barnhart et al., 2015). The escape latency time acquired after spatial navigation training reflects the learning capacity. A short ELT indicates strong learning capacity; conversely, a long ELT indicates poor learning capacity. As anticipated, 5xFAD mice showed marked impairment in spatial learning and memory. However, medium and high doses of BZD significantly ameliorated this deficit.

One of the most critical pathological features of AD is accumulation of Aβ, which is derived from APP through sequential cleavage by β-secretase and γ-secretase (Jiang et al., 2014). Here we showed that Aβ plaques were significantly reduced in the cortex and hippocampus of 5xFAD mice when treated with medium- and high-dose BZD, as well as donepezil HCl. Further, we showed that high-dose BZD decreased BACE1 and PS1 protein levels in 5xFAD mice, indicating that reduction of Aβ may be due to altered APP processing.

Increased activity of SOD typically indicates enhanced protection against oxidative stress and damage. However, at early stages of the inflammatory reaction (compensation stage), SOD activity is known to increase owing to the overproduction of free radicals. As anticipated, we found increased levels of MDA and higher SOD activity in the 5xFAD mice, suggesting that although these animals may suffer from damage caused by oxidative stress, they are likely to remain in the compensatory period. Interestingly, medium and high doses of BZD attenuated both MDA levels and SOD activity, suggesting beneficial effects on hippocampal oxidative stress.

Our results show the potential preventive effects of BZD during an early stage of AD (6-month-old 5xFAD mice), which may not be generalized to include later stages of the disease, where the pathology is already fully established and is more severe. Further studies evaluating the extent of benefits of BZD in more advanced stages of the disease are warranted, to fully unravel its therapeutic potential in AD. In addition, future studies are required to directly compare different therapeutic schemes that vary in the timing of administration, and to determine the best time-window for BZD administration. This knowledge may aid in designing future clinical studies to evaluate whether BZD is a viable treatment option in AD.

## Data Availability Statement

The data used to support the findings of this study are available from the corresponding authors upon request.

## Ethics Statement

All animal procedures were approved by the Laboratory Animal Management and Ethics Committee of Xiamen University, and the Animal Ethics Committee Guidelines of the Animal Facility of the Zhongshan Hospital Xiamen University.

## Author Contributions

YH and XZha conceived and designed the experiments, AP, XZhu, YG, YL, WH, SX, YW, WC, XH, TC, and RY performed the experiments, AP and YG analyzed the data, AP and XZhu prepared the manuscript, and SX wrote the contents and constructed the figures involved UHPLC-MS, and critically revised this manuscript as well as XZha. All authors have read and approved the manuscript.

## Conflict of Interest

The authors declare that the research was conducted in the absence of any commercial or financial relationships that could be construed as a potential conflict of interest.
