# Supplementary material for: Bazhu Decoction, a Traditional Chinese Medical Formula, Ameliorates Cognitive Deficits in the 5xFAD Mouse Model of Alzheimer’s Disease
Source: Front Pharmacol. 2019 Nov 27;10:1391. doi: 10.3389/fphar.2019.01391 (PMC6890723; doi:10.3389/fphar.2019.01391)
Supplement: Supplementary file 1 [file Table_1.docx]

**SUPPLEMENTARY TABLE 1∣**Information of components in BZD identified by UHPLC.

| Herb | Structure/Name (no.) | FW | MW | ESI ion peaks in Herb | | | ESI ion peaks in Formula 2 | | | | |
| --- | --- | --- | --- | --- | --- | --- | --- | --- | --- | --- | --- |
|  |  |  |  | tR (min) | ESI^+^ * | ESI^-^ | tR (min) | ESI^+^ * | ESI^-^ | References | |
| Morindae  Officinalis  Radix |   Asperuloside (**M1**) | C18H22O11 | 414.36 | 8.82 |  | 413.1089 | 8.80 |  | 413.1094 | (Zhao et al., 2018a) | |
|  |   rubiadin (**M2**) | C15H10O4 | 254.24 | 20.86 |  | 253.0510 | 20.87 |  | 253.0511 | | (Wang et al., 2017; Cui et al., 2014) |
|  |   physcion (**M3**) | C16H12O5 | 284.27 | 21.31 |  | 283.0613 | 21.36 |  | 283.0614 | | (Zhao et al., 2018b) |
|  |   rubiadin-1-methyl ether (**M4**) | C16H12O4 | 268.26 | 22.94 |  | 267.0664 | 22.93 |  | 267.0665 | | (Cui et al., 2014) |
|  |   1,6-dihydroxy-2-methoxyanthraquinone  (**M5**) | C15H10O5 | 270.24 | 24.21 |  | 269.0462 | 24.34 |  | 269.0462 | | (Wang et al., 2017) |
| Grassleaf  Sweelflag  Rhizome |   2,4,5-trimethoxybenzaldehyde (**G1**) | C10H12O4 | 196.20 | 16.31 | 197.0812 |  | 16.32 | 197.0813 |  | | (Li et al., 2019; Ni and Yu, 2013) |
|  |   β-asarone (**G2**) | C12H16O3 | 208.25 | 16.99 | 209.1173 |  | 16.97 | 209.1174 |  | | (McGaw et al., 2002) |
| Fructus Corni |   5-hydroxymethyl-2-furaldehyde (**C1**) | C6H6O3 | 126.11 | 6.23 | 127.0393 |  | 6.23 | 127.0394 |  | | (Yu et al., 2011) |
|  |   morroniside (**C2**) | C17H26O11 | 406.38 | 7.96 | 429.1366 | 405.1409 | 7.88 | 405.1411 | 429.1368 | | (Tao et al., 2018; Xue et al., 2014; Yu et al., 2011) |
|  |   loganin (**C3**) | C17H26O10 | 390.38 | 9.14 | 413.1410 | 389.1464 | 9.18 | 413.1411 | 389.1466 | | (Tao et al., 2018; Xue et al., 2014; Yu et al., 2011) |
|  |   sweroside (**C4**) | C16H22O9 | 358.34 | 9.60 | 359.1335, 381.1151 | 357.1189 | 9.61 | 359.1339, 381.1155 | 357.1191 | | (Chen and Ma, 2005) |
|  |   cornuside (**C5**) | C24H30O14 | 542.49 | 12.81 | 565.1521 | 541.1555 | 12.82 | 565.1529 | 541.1557 | | (Wang et al., 2017) |
| Arisaema  Cum  Bile |   taurochenodeoxycholic acid (**A1**) | C26H45NO6S | 499.69 | 14.43 |  | 498.2907 | 14.92 |  | 498.2909 | | (Liu et al., 2018) |
|  |   glycohyodeoxycholic acid (**A2**) | C26H43NO5 | 449.63 | 14.85 | 472.3051 | 448.3070 | 15.29 |  | 448.3070 | | (Liu et al., 2018; Cai et al., 2016) |
|  |   cholic acid (**A3**) | C24H40O5 | 408.57 | 20.21 |  | 407.2807 | 20.19 |  | 407.2807 | | (Liu et al., 2018; Cai et al., 2016) |
|  |   hyodeoxycholic acid (**A4**) | C24H40O4 | 392.58 | 22.28 |  | 391.2856 | 22.50 |  | 391.2856 | | (Liu et al., 2018) |
| Rainworm |   leucine (**R1**) | C6H13NO2 | 131.18 | 3.76 | 132.1021 | 130.0862 | 3.77 | 132.1022 | 130.0863 | | (Zhang et al., 2017) |
|  |   adenine (**R2**) | C5H5N5 | 135.13 | 4.92 | 136.0620 | 134.0463 | 4.87 | 136.0621 | 134.0460 | | (Zhang et al., 2017) |
|  |   phenylalanine (**R3**) | C9H11NO2 | 165.19 | 5.27 | 166.0862 | 164.0706 | 5.29 | 166.0863 | 164.0707 | | (Zhang et al., 2017) |
|  |   tryptophan (**R4**) | C11H12N2O2 | 204.23 | 6.76 | 205.0971 | 203.0822 | 6.75 | 205.0972 | 203.0823 | | (Zhang et al., 2017) |

* [M+H]^+^ or [M+Na]^+^.

**REFERENCES**

Zhao, X.S., Gong, B., Zhou, Y.K., and Yang, M.H. (2018a). Simultaneous determination of four iridoid glycosides in Morindae Officinalis Radix by UPLC-MS/MS. *Chin. J. Pharm. Anal.* 38, 1490-1945. doi: 10.16155/j.0254-1793.2018.09.04

Cui, N., Shi, J., and Jia, T.Z. (2014). Research on HPLC fingerprints of different processed Morinda officinalis. *Chin. Tradit. Herbal Drugs* 45, 1871-1875. doi: 10.7501/j.issn.0253-2670.2014.13.012

Cai, Y.Y., Ye, Y.H., Yang, L.Y., Huang, Y.M., Wu, Z.J., Ma, Y.D., et al. (2016). Determination of cholic acid constituents from Arisaema cum Bile by HPLC-ELSD. *J. Guangdong Pharm. Univ.* 32, 311-314. doi: 10.16809/j.cnki.1006-8783.2016032301

Chen, J., and Ma, S.C. (2005). HPLC determination of Sweroside and loganin in Caulis lonicerae. *Chin. J. Pharm. Anal.* 25, 1451-1452. doi: 10.16155/j.0254-1793.2005.12.004

Li, J., Chen, L., Ma, S.S., Wang, Z.Y., Zhou, Q.Y., Yi, L.Z., et al. (2019). Study on the chemical characteristics from different parts of Acorus tatarinowii by UPLC-HRMS combined with chemometrics. *J. Chin. Med. Mat*. 42, 1306-1311. doi: 10.13863/j.issn.1001-4454.2019.06.019

Liu, X.Y., Tao, X., Pan, D., Gao, H., Jia, T.Z., and Xu, D. (2018). Chemical constituents from Arisaema cum Bile. *Chin. Tradit. Patent Med*. 40, 1991-1995. doi: 10.3969/j.issn.1001-1528.2018.09.020

McGaw, L.J., Jäger, A.K., van Staden, J., Eloff., J.N. Isolation of β-asarone, an antibacterial and anthelmintic compound, from Acorus calamus in South Africa. *S. Afr. J. Bot*. 68, 31-35. doi: 10.1016/s0254-6299(16)30450-1

Ni, G., and Yu, D.Q. Chemical constituents from rhizomes of Acorus tatarinowii. *China J. Chin. Mat. Med*. 38, 569-573. doi: 10.4268/cjcmm20130420

Tao, J.H., Zhao, M., Jiang, S., Pu, X.L., and Wei, X.Y. (2018). Comparative metabolism of two major compounds in Fructus Corni extracts by gut microflora from normal and chronic nephropathy rats in vitro by UPLC-Q-TOF/MS. *J. Chromatogr. B* 1073, 170-176. doi: 10.1016/j.jchromb.2017.12.025

Wang, M.L., Zhang, Q.Q., Fu, S., Liu, Y.H., Liang, C.L., Chen, N., et al. (2017). Characterization of Morinda officinalis How. By UPLC-Q-TOF MS^E^ coupled with UNIFI database filter. *J. Chin. Mass Spectr. Soc.* 38, 75-82. doi: 10.7538/zpxb.2017.38.01.0075

Wang, X.Y., Huo, T.T., and Li, Z.G. (2017). Simultaneous determination of 4 active components in Qijudihuang oral liquid by QAMS method. *Chin. J. Pharm. Anal.* 37, 290-296. doi: 10.16155/j.0254-1793.2017.02.15

Xue, C., Zhang, A., Sun, H., Han, Y., Zou, D., Wang, Y., et al. (2014). [An improved ultra-performance liquid chromatography-electrospray ionization/quadrupole-time-of-flight high-definition mass spectrometry method for determining ingredients of herbal Fructus corni in blood samples](http://med.wanfangdata.com.cn/Paper/Detail/PeriodicalPaper_PM25422541). *Pharmacogn. Mag.* 10, 422-429. doi: 10.4103/0973-1296.141796

Yu, X.H., Bi, K.S., Li, Z.Y., Dai, R.H., and Chen, X.H. (2011). UPLC simultaneous determination of five components in crude and wine treat Fructus Corni. *Chin. J. Pharm. Anal.* 31, 1463-1466. doi: 10.16155/j.0254-1793.2011.08.008

Zhao, Q., Chen, Y.P., Cui, X.S., Tian, Q.C., Shen, S., Bi, D., et al. (2018b). Study on multi-compound determination and fingerprint of Rheum palmatum by ultra performance liquid chromatography. *Chin. J. Pharm. Anal.* 38, 1697-1710. doi: 10.16155/j.0254-1793.2018.10.06

Zhang, Y., Dong, W.T., Huo, J.H., and Wang, W.M. (2017). Analysis on chemical constituents of Pheretima aspergillum by UPLC-Q-TOF-MS. *Chin. Tradit. Herbal Drugs* 48, 252-262. doi: 10.7501/j.issn.0253-2670.2017.02.006
